# Caffeine administration prevents retinal neuroinflammation and loss of retinal ganglion cells in an animal model of glaucoma

**DOI:** 10.1038/srep27532

**Published:** 2016-06-08

**Authors:** Maria H. Madeira, Arturo Ortin-Martinez, Francisco Nadal-Nícolas, António F. Ambrósio, Manuel Vidal-Sanz, Marta Agudo-Barriuso, Ana Raquel Santiago

**Affiliations:** 1 Institute for Biomedical Imaging and Life Sciences (IBILI), Faculty of Medicine, University of Coimbra, 3000-548 Coimbra, Portugal; 2 CNC.IBILI, University of Coimbra, 3004-504 Coimbra, Portugal; 3 Departamento de Oftalmología, Facultad de Medicina, Universidad de Murcia & Instituto Murciano de Investigacion Biosanitaria Hospital Virgen de la Arrixaca (IMIB-Arrixaca), 30100 Murcia, Spain; 4 Association for Innovation and Biomedical Research on Light and Image (AIBILI), 3000-548 Coimbra, Portugal

## Abstract

Glaucoma is the second leading cause of blindness worldwide, being characterized by progressive optic nerve damage and loss of retinal ganglion cells (RGCs), accompanied by increased inflammatory response involving retinal microglial cells. The etiology of glaucoma is still unknown, and despite elevated intraocular pressure (IOP) being a major risk factor, the exact mechanisms responsible for RGC degeneration remain unknown. Caffeine, which is an antagonist of adenosine receptors, is the most widely consumed psychoactive drug in the world. Several evidences suggest that caffeine can attenuate the neuroinflammatory responses and afford protection upon central nervous system (CNS) injury. We took advantage of a well characterized animal model of glaucoma to investigate whether caffeine administration controls neuroinflammation and elicits neuroprotection. Caffeine or water were administered *ad libitum* and ocular hypertension (OHT) was induced by laser photocoagulation of the limbal veins in Sprague Dawley rats. Herein, we show that caffeine is able to partially decrease the IOP in ocular hypertensive animals. More importantly, we found that drinking caffeine prevented retinal microglia-mediated neuroinflammatory response and attenuated the loss of RGCs in animals with ocular hypertension (OHT). This study opens the possibility that caffeine or adenosine receptor antagonists might be a therapeutic option to manage RGC loss in glaucoma.

Glaucoma is a group of progressive neurodegenerative multifactorial diseases, characterized by the loss of retinal ganglion cells (RGCs), optic nerve excavation, and axonal degeneration leading to irreversible vision loss^1^. Although the etiology of glaucoma is still not completely elucidated, advanced age and elevation of intraocular pressure (IOP) are considered the main risk factors for the disease onset. Current available treatments for glaucoma are focused on the reduction of IOP, the only modifiable risk factor^2^. However, in several patients the disease still progresses, despite the effective control of IOP. Therefore, it is urgent to develop novel therapeutic strategies focused on the neuroprotection of RGCs^3^.

It is currently recognized that degeneration of RGCs in human and experimental glaucoma is accompanied by a neuroinflammatory response, involving retinal microglial cells and increased production of inflammatory mediators, such as tumor necrosis factor (TNF) and interleukin-1β (IL-1β)^4^,^5^,^6^,^7^. In addition, early and exacerbated activation of retinal microglial cells has been described and proposed to contribute to the degenerative process^8^,^9^,^10^, suggesting that the control of microglia reactivity can prevent the glaucomatous loss of RGCs^11^,^12^,^13^.

The adenosine A_2A_ receptor (A_2A_R) is an important drug target in the central nervous system (CNS), since its blockade has been shown to afford robust neuroprotection in different noxious brain conditions, namely through the control of microglia-mediated neuroinflammatory processes^14^. Recently, we showed that the blockade of the A_2A_R affords protection to RGCs against damage induced by elevated hydrostatic pressure in retinal organotypic cultures^15^ as well as in the high IOP-induced transient ischemic injury animal model^16^. We also demonstrated that A_2A_R blockade prevents retinal microglia reactivity and the associated neuroinflammatory response, suggesting the control of microglia-mediated neuroinflammation as the mechanism operated by A_2A_R antagonist to provide retinal protection^16^.

Caffeine is the most widely consumed psychoactive drug in the world. In the CNS, the effects exerted by caffeine, at non-toxic doses, are mediated through the antagonism of adenosine receptors^17^. Caffeine, by blocking A_2A_R, is able to prevent synaptotoxicity, excitotoxicity and neuronal loss^18^,^19^,^20^,^21^. In addition, it has also been reported that caffeine has anti-inflammatory properties in the CNS^22^, namely by attenuating microglia-mediated neuroinflammation^23^.

Taking in consideration the neuroprotective properties of caffeine in the brain mediated by A_2A_R blockade, together with our previous studies, we now hypothesize that caffeine may confer neuroprotection to RGCs in models of glaucoma by controlling the neuroinflammatory response.

Therefore, the main aim of this work was to investigate whether caffeine administration modulates retinal neuroinflammation and prevents the loss of RGCs in an animal model (Sprague Dawley rats) of ocular hypertension (OHT), obtained by laser photocoagulation (LP) of the trabecular meshwork and limbal veins. Although this model does not completely mimic human glaucomatous optic neuropathy, it has been extensively used to evaluate anatomical and functional alterations associated with glaucomatous damage, such as loss of RGCs and impairment of the retrograde axonal transport in the optic nerve^24^,^25^,^26^,^27^.

## Results

Ocular hypertension induced by LP of the limbal and episcleral vessels of adult rats triggers anatomical and functional alterations associated with glaucoma, such as loss of RGCs and impaired retrograde axonal transport of the optic nerve^24^,^25^. We took advantage of this animal model of glaucoma to investigate the ability of caffeine to modulate retinal neuroinflammatory response and evaluate its neuroprotective role.

### Effect of caffeine consumption in animal weight, fluid intake and IOP

Caffeine (1 g/L) was administered in the drinking water, starting 2 weeks prior the induction of OHT and until the end of the study. Animal weight and fluid intake were registered in all animals during treatment (Table 1). No significant alterations were observed in the fluid intake or weight between animals drinking water or caffeine.

**Figure 1:**
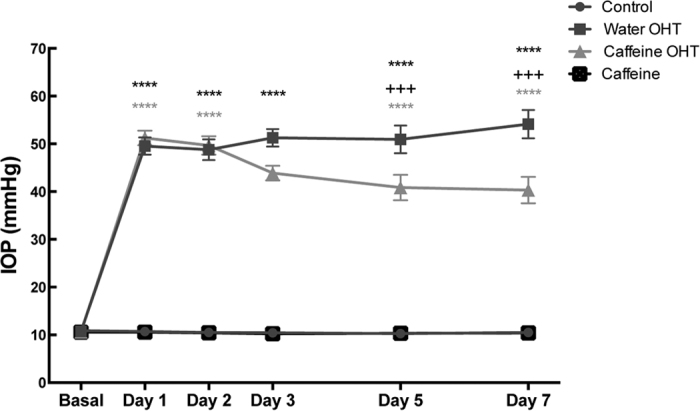
Caffeine administration reduces IOP in OHT animals. Water or caffeine (1 g/L) was administrated *ad libitum* to Sprague Dawley rats, during 2 weeks prior induction of OHT, and until the end of the experiment. IOP was measured with a rebound tonometer. Results are expressed in mmHg and represent the mean ± s.e.m of 22 to 37 independent experiments. ****p < 0.0001, significantly different from control animals; ^+++^p < 0.001, significantly different from OHT control animals; Two-way ANOVA, followed by Tukey’s multiple comparison test.

Since caffeine consumption may change IOP^28^, IOP was measured in all animals prior inducing OHT (basal) and at days 1, 2, 3, 5 and 7 post-OHT induction with a rebound tonometer (Fig. 1). The basal IOP was similar in all groups (as reference, IOP in control animals was 10.8 ± 0.2 mmHg, n = 22). As expected, 1 day post-OHT induction IOP significantly increased in both water- and caffeine-drinking animals (49.5 ± 1.8 and 51.2 ± 1.5 mmHg, respectively, n = 37; p < 0.0001, when compared with basal IOP). In these animals, IOP maintained elevated throughout the experiment. Nevertheless, 3 days post-OHT induction, the IOP in caffeine-drinking animals with OHT was statistically lower, when compared with the water-drinking animals subjected to OHT (43.9 ± 1.5 mmHg and 51.2 ± 1.8 mmHg, respectively; p < 0.001). This effect was maintained until the end of the study, and at day 7 post-OHT induction, IOP of water-drinking animals with OHT was 54.1 ± 2.5 mmHg and in caffeine-drinking animals with OHT the IOP was 40.3 ± 2.8 mmHg (p < 0.001). Caffeine administration per se did not alter the IOP.

### OHT increases the expression of A_2A_R and does not affect A_1_R expression

**Figure 2:**
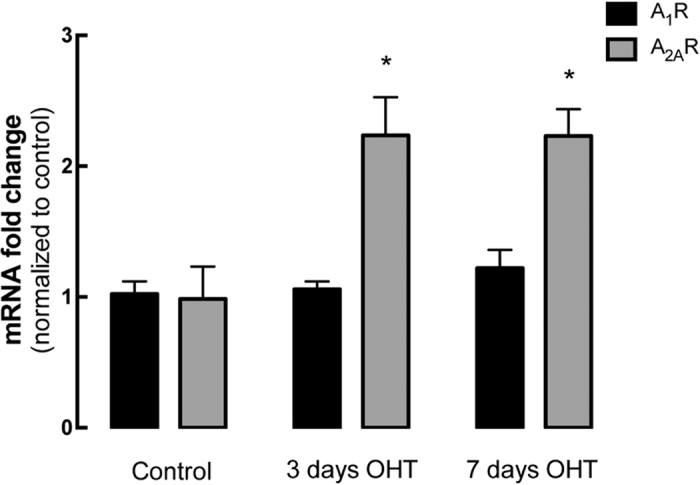
OHT induces up-regulation of A_2A_R without altering the expression of A_1_R. The expression of A_1_R and A_2A_R mRNA was assessed by qPCR in the retinas of control animals and animals with 3 or 7 days of OHT. Results are presented as fold change comparing with the control animals, and represent the mean ± s.e.m of 5 to 7 independent experiments. ^#^p < 0.05, significantly different from control animals; Kruskall-Wallis test, followed by Dunn’s multiple comparison test.

In the CNS, including the retina, the pharmacological actions of caffeine are exerted mainly by antagonizing adenosine receptors^29^. It has been documented that brain noxious conditions downregulate A_1_Rs and up-regulate A_2A_Rs. Therefore, we evaluated whether OHT could alter the expression of A_1_Rs and A_2A_Rs. No significant alterations were detected in the mRNA expression of A_1_R (Fig. 2) at the 2 time-points analyzed (3 and 7 days after inducing OHT). Nevertheless, at 3 days post-OHT we detected a significant increase in the expression of A_2A_R compared with control animals (2.3-fold change; n = 5, p < 0.05), which was maintained until 7 days post-OHT (2.4-fold change; n = 7, p < 0.05).

### Caffeine inhibits the inflammatory response triggered by OHT

**Figure 3:**
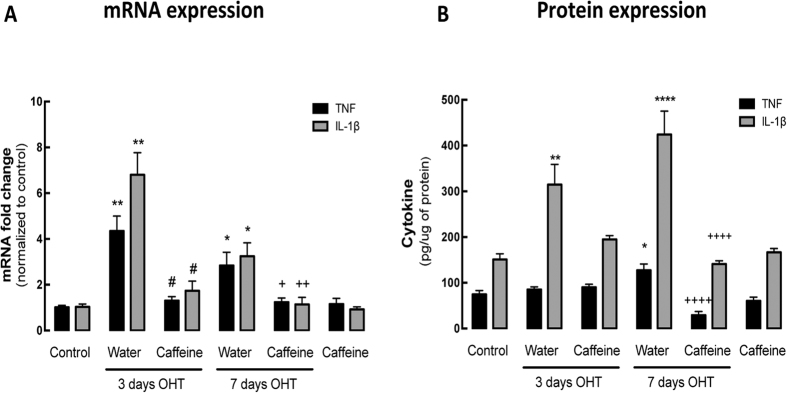
Caffeine administration prevents the inflammatory response triggered by OHT. The effects of caffeine administration on the retinal neuroinflammatory response in eyes subjected to OHT were evaluated by qPCR and ELISA. (**A**) mRNA expression of pro-inflammatory cytokines IL-1β and TNF were assessed by qPCR. Results are presented as fold change comparing with the control animals, and represent the mean ± s.e.m from 5–7 independent experiments. (**B**) The retinal protein levels of IL-1β and TNF were quantified by ELISA. Results are expressed in pg/μg of protein, and represent the mean ± s.e.m from 5–10 independent experiments. *p < 0.05, **p < 0.01 and ****p < 0.0001, significantly different from control animals; ^#^p < 0.05, significantly different from water drinking animals with 3 days OHT; ^+^p < 0.05, ^++^p < 0.01 and ^++++^p < 0.0001, significantly different from water drinking animals with 7 days OHT; Kruskall-Wallis test, followed by Dunn’s multiple comparison test.

Since chronic inflammation plays an important role in the pathophysiology of glaucoma^30^, we evaluated mRNA and protein levels of the pro-inflammatory markers TNF and IL-1β. As shown in Fig. 3A, 3 days with OHT significantly increased the mRNA levels of TNF and IL-1β (4.3 ± 0.6 and 6.8 ± 0.9 fold-change of the control, respectively; n = 7, p < 0.01). Similarly, at 7 days with OHT the mRNA levels of these cytokines were still significantly elevated, when compared with the control (2.8 ± 0.6 and 3.2 ± 0.3 fold change for TNF and IL-1β, respectively; n = 7, p < 0.05). The administration of caffeine to OHT animals significantly inhibited the OHT-induced up-regulation of TNF and IL-1β mRNA levels (n = 7, p < 0.05) in both time points, without altering the expression of both TNF and IL-1β in the retinas of water-drinking control animals (control group).

We then quantified the protein levels of TNF and IL-1β, by ELISA, in retinal extracts (Fig. 3B). In the retinas of control animals, the expression of TNF and IL-1β was 74.6 ± 8.6 and 150.5 ± 12.6 pg/μg of total protein, respectively. At 3 and 7 days after inducing OHT, IL-1β protein levels were significantly increased (314.7 ± 44.3 and 424.1 ± 51.2 pg/μg of total protein, respectively; n = 8–9, p < 0.01 and p < 0.001), when compared with control animals. In animals drinking caffeine, after 3 days of OHT, the levels of IL-1β in the retina slightly decreased to 194.9 ± 8.1 pg/μg of total protein, comparing with animals drinking water. Moreover, at 7 days of OHT the administration of caffeine significantly decreased IL-1β to 140.9 ± 7.1 pg/μg of total protein (n = 7, p < 0.001). After 3 days of OHT, the protein levels of TNF were not altered in retinal extracts both in water and caffeine drinking animals, comparing with control animals. Nevertheless, after 7 days of OHT the protein levels of TNF significantly increased to 127.4 ± 13.7 pg/μg of total protein (n = 9, p < 0.05), which was prevented by caffeine administration (29.9 ± 7.98 pg/μg protein; n = 8, p < 0.0001). Caffeine administration, per se, did not alter the protein levels of these two cytokines.

### Caffeine prevents OHT-induced microglia activation

Microglia activation appears early in the retina of animal models of glaucoma^9^, implicating a role for these cells in the early stages of the disease^30^,^31^. Therefore, we evaluated whether caffeine administration could modulate microglia reactivity elicited by OHT.

**Figure 4:**
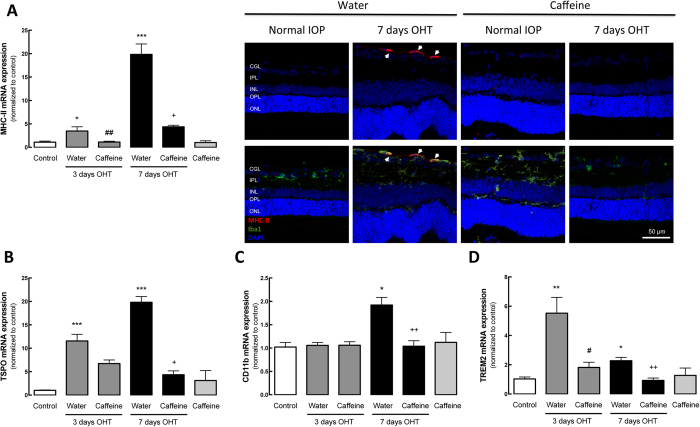
Caffeine prevents OHT-induced microglia reactivity. Effects of caffeine administration on the mRNA expression of microglial cell markers. (**A**) MHC-II, (**B**) TSPO, **(C)** CD11b and **(D)** TREM2 mRNA levels were assessed by qPCR. Results are presented as fold change comparing with the control, from 5–7 independent experiments. **(E)** Retinal sections were immunostained for Iba1 (general microglia marker; green) and MHC-II (activated microglia marker; red) and then were imaged in a confocal microscope. Nuclei were stained with DAPI (blue). Representative images obtained from 5 independent experiments. Results are presented as fold change comparing with the control, and represent the mean ± s.e.m from 5–7 independent experiments. *p < 0.05, **p < 0.01 and ***p < 0.001, significantly different from control; ^#^p < 0.01, significantly different from water drinking animals with 3 days OHT; ^+^p < 0.05, ^++^p < 0.01 and ^++++^p < 0.0001, significantly different from water drinking animals with 7 days OHT; Kruskall-Wallis test, followed by Dunn’s multiple comparison test.

By qPCR, we evaluated the mRNA levels of two markers of microglia reactivity, major histocompatibility complex class II (MHC-II) (Fig. 4A) and translocator protein (18 kDa) (TSPO)^32^,^33^,^34^ (Fig. 4B), as well as CD11b, a general marker of microglia (Fig. 4C) and triggering receptor expressed on myeloid cells 2 (TREM2), which is associated with microglia phagocytic capacity (Fig. 4D). After 3 and 7 days of OHT, there was a significant increase in the mRNA levels of MHC-II (3.5 ± 0.8 and 19.8 ± 2.2 fold change, respectively; n = 7, p < 0.05 and p < 0.001). Caffeine administration significantly prevented the OHT-induced increase in MHC-II expression (1.2 ± 0.8 and 4.4 ± 0.3 fold change for 3 and 7 days of OHT, respectively; n = 7, p < 0.05 and p < 0.01). The immunoreactivity for MHC-II was mainly detected in the ganglion cell layer in OHT animals and co-localized with Iba1, a general marker of microglia (Fig. 4E). In accordance with the qPCR results, MHC-II immunoreactivity in the retina was increased in animals with OHT for 7 days; an effect that was not observed in the caffeine-drinking OHT animals.

The mRNA expression of TSPO (Fig. 4B) was also up-regulated after 3 and 7 days of OHT (11.6 ± 1.5- and 19.8 ± 1.2-fold change, respectively; n = 7, p < 0.001). The administration of caffeine slightly decreased the levels of the mRNA coding for TSPO (6.7 ± 0.8-fold change) at 3 days in OHT animals, and the decrease reached statistical significance at 7 days in animals with OHT drinking caffeine (4.3 ± 0.8-fold change; n = 7, p < 0.05), compared with water drinking animals.

We then assessed the expression levels of CD11b, and detected a significant up-regulation of the mRNA levels after 7 days of OHT (1.9 ± 0.2-fold change; n = 7, p < 0.05). This up-regulation was prevented by caffeine administration (1.1 ± 0.2-fold change; n = 7, p < 0.01). The expression of TREM2 was also assessed in order to estimate microglia phagocytic activity. OHT for 3 and 7 days significantly increased the expression of TREM2 mRNA by 5.5 ± 1.0 and 2.3 ± 0.32 fold-change, respectively (n = 7, p < 0.05 and p < 0.01), and caffeine prevented the OHT-induced increase in TSPO expression (1.8 ± 0.4- and 0.9 ± 0.2-fold change, for 3 and 7 days, respectively; n = 7, p < 0.05 and p < 0.01). Administration of caffeine per se did not alter the expression of pro-inflammatory markers.

### Caffeine administration prevents microglia reactivity in contralateral retina induced by OHT

Microglia reactivity in the contralateral eye (without OHT) has been reported^35^,^36^. Therefore, we evaluated the ability of caffeine to modulate the microglia reactivity in contralateral eyes.

**Figure 5:**
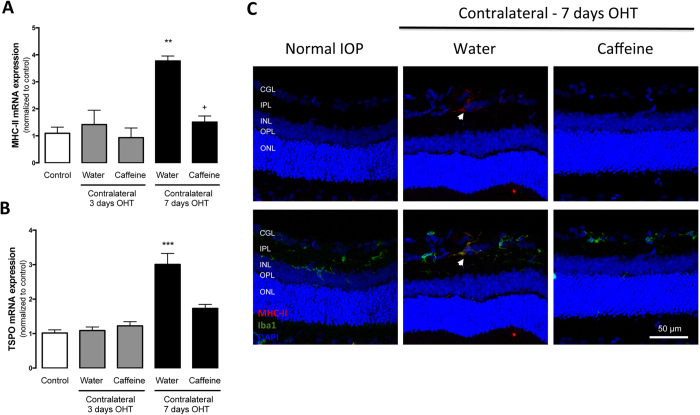
Caffeine prevents OHT-induced microglia reactivity in the contralateral eye. Effect of caffeine administration on OHT-induced microglia activation in the contralateral eye was evaluated by qPCR to assess mRNA expression of microglial cell activation markers MHC-II (**A**) and TSPO (**B**). Results are presented as fold change comparing with the control, from 5–7 independent experiments. (**C**) Retinal sections were immunostained for Iba1 (general microglial marker; green) and MHC-II (activated microglia marker; red) and then were imaged in a confocal microscope. Nuclei were stained with DAPI (blue). Representative image obtained from 5 independent experiments. Results are presented as fold change comparing with the control, and represent the mean ± s.e.m from 5–7 independent experiments. **p < 0.01 and ***p < 0.001, significantly different from control; ^+^p < 0.05, significantly different from the contralateral eye of water drinking animals with 7 days OHT; Kruskall-Wallis test, followed by Dunn’s multiple comparison test.

The expression of mRNA coding for MHC-II and TSPO (Fig. 5A,B) in the contralateral eye was significantly up-regulated after 7 days of OHT (3.8 ± 0.2- and 3.0 ± 0.3-fold change, p < 0.01 and p < 0.001, respectively; n = 7). Caffeine administration reduced the expression of MHC-II and TSPO in the contralateral eyes, significantly for MHC-II (1.5 ± 0.2-fold change, p < 0.05; n = 6). By immunohistochemistry, similarly to the OHT eyes, MHC-II immunoreactivity is detected in the microglia (Iba1-positive cells) within the GCL (Fig. 5C). Caffeine administration inhibited the OHT-induced increase in MHC-II immunoreactivity in the contralateral eyes.

### Caffeine administration does not ameliorate the OHT-induced impairment in the RGC retrograde transport

**Figure 6:**
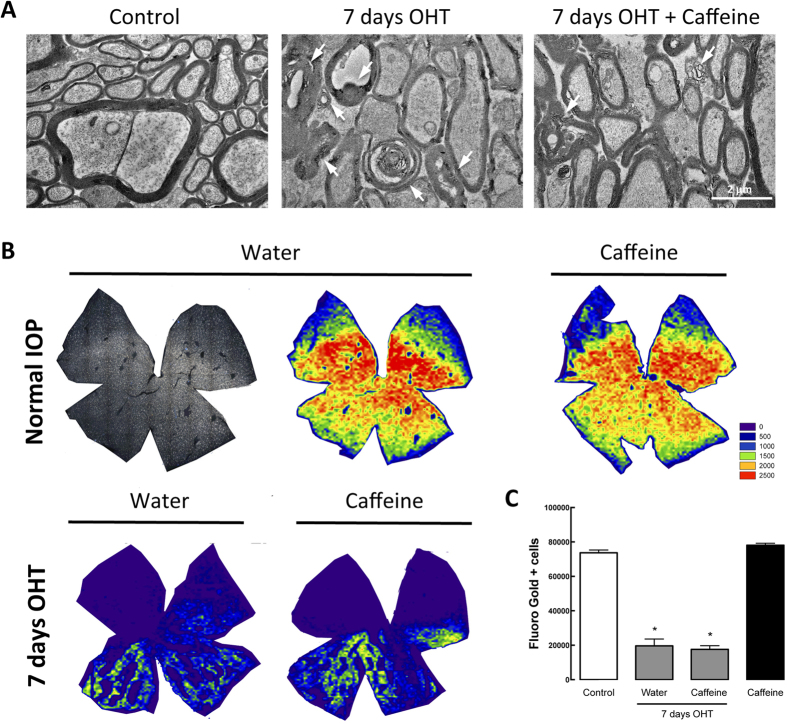
Caffeine partially prevents OHT-induced optic nerve structural alterations but does not improve axonal transport impairment. (**A**) Optic nerve structural alterations were observed by transmission electron microscopy. Representative images of semi-thin cross-sections of control, and water drinking and caffeine drinking animals subjected to 7 days of OHT. Alterations in axons structural, including degenerating axons and myelin disarrangement (arrows) can be observed in OHT animals. Scale bar: 2 μm. (**B**) Retrograde axonal transport was assessed by FG application in the superior colliculus 1 day after induction of OHT, and whole-mounted FG-labeled retinas were imaged. Representative isodensity maps showing the topological distribution of FG-positive RGCs, using a color code, according to cell density value within a 28-step color scale range from 0 (dark blue) to 2500 or higher RGCs/mm^2^ (red). (**C**) Quantification of FG-positive cells. Graph represents mean ± s.e.m. of the number of FG-positive cells, from 5 to 7 independent experiments. *p < 0.05, significantly different from control; Kruskall-Wallis test, followed by Dunn’s multiple comparison test.

Alterations in the structure of the optic nerve and in the retrograde axonal transport of RGCs have been described in glaucomatous animal models^24^,^37^. Therefore, in OHT animals, we assessed the effect of caffeine administration in both the integrity of the optic nerve and the axonal transport in RGCs. The structural integrity of the optic nerve was assessed by transmission electron microscopy (Fig. 6A). We observed that OHT increased the incidence of axon degenerative profiles, with disorganized and abnormal myelin wrapping (Table 2). Interestingly, in animals drinking caffeine this effect appears to be partially attenuated, with caffeine-drinking OHT animals presenting a reduced number of disorganized myelin structures (Fig. 6A and Table 2).

Retrograde axonal transport was assessed after Fluorogold (FG) application in both superior colliculi, the targets of 98% of RGCs^38^. Application of FG after induction of OHT is an established method to evaluate the impairment of the retrograde axonal transport of RGC, by counting the total number of FG-positive cells in whole-mounted retinas (FG^+^-labeled RGCs)^26^. Isodensity maps allowed us to visualize the distribution of FG^+^-RGCs in the retina (Fig. 6B). In control animals, drinking water or caffeine, the total number of FG^+^ cells was 73,698 ± 1,611 and 78,125 ± 1,096 cells, respectively (n = 5) (Fig. 6C), similar to previous reports^24^. This number was significantly reduced to 19,619 ± 3,990 cells (n = 4; p < 0.05) in animals with OHT for 7 days. In animals with OHT, caffeine administration did not prevent the OHT-induced reduction in the number of FG^+^ -labeled RGCs (17,630 ± 2,102 cells; n = 4, p < 0.05).

### Caffeine increases RGC survival in OHT animals

**Figure 7:**
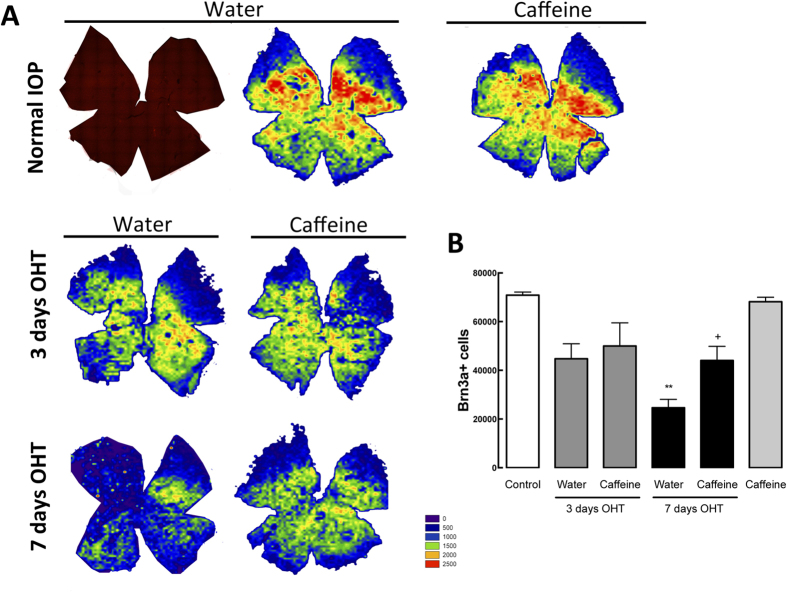
Caffeine administration inhibits Brn3a-positive cell loss triggered by OHT. Retinal whole-mounts were immunostained for Brn3a (red; RGC marker) and isodensity maps were generated to evaluate RGC survival. (**A**) Representative isodensity maps demonstrating the topological distribution of Brn3a-labelled RGCs, using a color code according to cell density value within a 28-step color scale range from 0 (dark blue) to 2500 or higher RGCs/mm^2^ (red). (**B**) The graph represents mean ± s.e.m. of the number of Brn3a-positive cells, from 5 to 7 independent experiments. **p < 0.01, significantly different from control; ^+^p < 0.05, significantly different from water drinking animals with 7 days OHT; Kruskall-Wallis test, followed by Dunn’s multiple comparison test.

Taking into consideration the protective properties of caffeine administration^39^, we evaluated the potential protective effect of caffeine against the degeneration of RGCs triggered by OHT. In whole-mounted retinas, RGCs were labeled with an antibody that recognizes Brn3a, a marker of RGCs^40^ (Fig. 7A), and the total number was counted automatically (Fig. 7B). In control animals, the total number of RGCs per retina was 70,861 ± 1,258 (n = 5), similarly to previous works^24^. The occurrence of OHT for 3 days triggered a reduction in the number of Brn3a-positive cells to 44,746 ± 6,151 cells (n = 4) and 50,021 ± 6,151 cells (n = 4) in animals drinking water and caffeine, respectively. Extension of OHT to 7 days resulted in a further significant loss of RGCs in animals drinking water (24,621 ± 3,443 Brn3a-positive cells, n = 7, p < 0.01). However, administration of caffeine to animals with OHT for 7 days significantly prevented the loss of RGCs induced by OHT (44,027 ± 5,841 Brn3a-positive cells, n = 7, p < 0.05). Caffeine administration to animals with normal IOP did not significantly alter the number of RGCs (68,169 ± 1,840 Brn3a-positive cells, n = 6).

## Discussion

The present work demonstrates that caffeine administration prevents retinal neuroinflammation, microglia reactivity and affords protection to RGCs in an animal model of glaucoma.

Similarly to what happens in chronic noxious brain conditions^41^,^42^, OHT triggered A_2A_R upregulation, prompting the hypothesis that the manipulation of this receptor may control neurodegeneration. We previously reported that elevated hydrostatic pressure, to mimic OHT *in vitro*, increases A_2A_R expression, mainly in retinal microglia located within the ganglion cell layer^15^. Since the actions of caffeine are exerted mainly by blocking adenosine receptors, including the high-affinity A_1_R and A_2A_R^17^, the up-regulation of A_2A_R by OHT suggested that the effects of caffeine in OHT animals were mediated by A_2A_R antagonism.

Caffeine affords beneficial effects in animal models of conditions expected to impair memory performance such as Parkinson’s disease, chronic stress, type 2 diabetes, attention deficit and hyperactivity disorder, early life convulsions, or alcohol-induced amnesia^21^. The effects of caffeine to ocular tissues have also been reported, like the prevention of diabetic cataract in galactose-fed animals and reduction of corneal thickness^43^. The effects of consumption of caffeine in IOP are not yet clarified. While some authors suggest that caffeine consumption may increase IOP in patients with normotensive glaucoma or ocular hypertension^28^, others have shown that caffeine does not significantly alter IOP in patients with glaucoma^44^. Therefore, we regularly measured IOP in animals and found that administration of caffeine was able to reduce the IOP of OHT animals, without interfering with animals with normal IOP. Nevertheless, the IOP lowering effect of caffeine may not be enough to explain the effects exerted by caffeine, since IOP in caffeine-drinking OHT animals is still elevated (30% increase above basal IOP^45^).

Glaucomatous damage is accompanied by early activation of microglia and increased expression of inflammatory mediators^6^,^8^,^9^,^12^,^46^,^47^,^48^. It has been suggested that the control of microglia reactivity may represent a therapeutic strategy to manage glaucoma. Reduction of microglia reactivity by irradiation or pharmacological treatment, or the reduction of TNF expression, confers protection in an animal model of glaucoma^11^,^12^,^49^. Several studies demonstrate that caffeine affords protection to the brain in models of neurodegenerative diseases^20^,^29^ and prevents microglia-mediated neuroinflammatory responses^23^. Indeed, OHT animals treated with caffeine presented reduced microglia activation and lower levels of inflammatory mediators, demonstrating that caffeine prevents microglia-mediated neuroinflammation induced by OHT. Increased expression of MHC-II has been also detected in mice contralateral eyes^35^,^36^. Remarkably, caffeine also reduced microglia reactivity in contralateral eyes (without OHT).

Although we did not assess the contribution of other cell types responsible for the inflammatory environment in the retina, we cannot discard the contribution of macroglial cells, which can also release inflammatory mediators^50^. Nevertheless, caffeine administration did not prevent OHT-induced GFAP up-regulation, a marker of astroglial and Müller cell reactivity (supplementary data; Fig. 1), suggesting that caffeine appears to be preferentially modulating microglia responses. This is in line with previous studies reporting the inability of A_2A_R to modulate the activation of astroglial cells^18^,^51^.

Several studies have shown that axonal transport in the optic nerve is impaired in human glaucoma and in the OHT rat model, preceding the loss of RGCs^24^,^26^,^38^,^52^. In this model, OHT results in Wallerian-like degeneration^53^, which culminates in RGC death^26^,^52^. In fact, previous studies have shown that part of the surviving RGC population after one week of OHT exhibits impaired retrograde axonal transport, supporting evidence that not all RGCs die immediately upon impaired axonal transport^25^,^26^.

In this work, we found that caffeine is not able to prevent the deficit in the axonal transport induced by OHT. Although caffeine-drinking animals with OHT presented a more preserved optic nerve structure, this was not sufficient to overcome the damage induced by OHT and it was not able to improve axonal transport. Nevertheless, there is a significant attenuation in the loss of RGCs induced by seven days of OHT in animals drinking caffeine. Glaucomatous injury to the optic nerve and optic nerve head appears to be related with the values of IOP, but the detailed mechanism remains to be elucidated^26^. In fact, previous reports using distinct models of IOP elevation have demonstrated that axonal damage in the optic nerve correlates with the magnitude and duration of IOP elevation^37^,^54^.

Although most of cell death occurs subsequently to the axonal degeneration, glial-mediated inflammatory response also contributes to the progress of the damage^55^. In fact, activation of microglia in a glaucoma animal model occurs prior the loss of RGCs^9^. In a model of LP-induced OHT, the presence of markers of microglia reactivity in the retina after 3 days of OHT is paralleled by a decrease in the number of RGCs^56^. Also, markers of reactive microglia are present in the optic nerve and optic tract after 7 days of induction of OHT^56^. Hence, caffeine, by blocking A_2A_R, might be attenuating microglia reactivity, thus protecting the soma of RGCs. Being the response of microglial cells in the optic nerve delayed, it seems plausible to speculate that the effects of caffeine might not be observed at the 7 days time-point. Also, adenosine receptors might not be directly related with the integrity of the retrograde axonal transport, and therefore caffeine might not be able to alter the functional damage induced by OHT. Indeed, as discussed above, functional impairment of retrograde axonal transport is directly correlated with the elevation of IOP^37^,^54^.

Notably, we have not observed any detrimental effect related with caffeine administration neither in animals with normal IOP nor with OHT. In fact, caffeine is able to reduce the neuroinflammatory response and increase the survival of RGCs in animals with OHT, in a mechanism independent of lowering IOP.

Herein, we demonstrate that caffeine consumption, as described in other neurodegenerative diseases, prevented the loss of RGCs associated with glaucoma. Taking in account the results obtained in this work, together with our previous works^15^,^16^, selective antagonists of A_2A_R, in combination with IOP lowering agents, might be envisaged as a potential therapeutic strategy to treat glaucoma.

## Methods

### Animals

All procedures involving animals were approved by the Ethical and Animal Studies Committee of the University of Murcia and were in accordance with the ARVO and European Union guidelines for the use of animals in research. Adult female albino Sprague Dawley rats, with 8 to 10 weeks, (Charles River Laboratories, L’Arbresle, France) were housed in the animal facilities of the University of Murcia, Spain, and were provided with standard rodent diet and water ad libitum, under a 12 h light/12 h dark cycle. Recent studies suggest that injury to one eye may produce significant molecular and structural changes in the intact contralateral eye^35^,^36^,^57^,^58^. Therefore, comparisons were performed using a group of control animals.

### Caffeine administration

Animals were randomly assigned to receive caffeine or normal drinking water. Caffeine (1 g/L, Sigma-Aldrich, St. Louis, MO, USA) was supplied in the drinking water for two weeks before the induction of OHT and was maintained until the end of the experiment. The chosen dose of caffeine was based in a previous a work that reported protective effects of caffeine intake^59^.

The animals were divided into 6 experimental groups: 1) control (intact and water drinking); 2) caffeine (normal IOP and caffeine drinking); 3) water drinking +3 days OHT; 4) caffeine drinking +3 days OHT; 5) water drinking +7 days OHT; 6) caffeine drinking +7 days OHT.

### Induction of OHT and IOP measurements

To avoid the IOP-lowering effect of anesthetic agents, IOP measurements were performed in non-sedated rats that were trained as previously described^60^. The IOP was measured in both eyes before surgery (basal) and at days 1, 2, 3, 5 and 7 post-surgery with a rebound tonometer specifically designed for rodents (Tonolab®, Icare, Espoo, Finland). IOP was considered elevated when the mean of IOP measurements collected increase by 30% compared with basal IOP^45^.

OHT was induced in the left eyes of anesthetized [intraperitoneal injection of xylazine (10 mg/kg) and ketamine (60 mg/kg)] rats in a single session of diode laser burns (Viridis Ophthalmic Photocoagulator-532 nm, Quantel Medical, Clermont-Ferrand, France), as we described previously^24^,^27^,^38^. During recovery, topical ointment containing Tobramycin was applied to the eyes to prevent corneal desiccation.

### Retrograde tracing of retinal ganglion cells

One day after laser photocoagulation, 3% fluorogold (FG; Fluorochrome Inc., Engelwood, CO, USA) diluted in 10% in DMSO saline was applied onto the surface of both superior colliculi (SCi), as previously described^24^,^38^,^61^. Retinas were analyzed 6 days after the tracing, 7 days after the induction of the OHT.

### Immunodetection

All animals were euthanized with an intraperitoneal overdose of 20% sodium pentobarbital and then transcardially perfused with saline followed by 4% (w/v) paraformaldehyde (PFA).

#### Flat-mounted retinas

Retinas from both eyes were dissected and then permeabilized with 0.5% Triton X-100 in PBS, followed by freezing at −70 °C for 15 min, and then a new rinse in 0.5% Triton X-100. Brn3a was detected by incubation with a goat anti-Brn3a antibody (1:750; C-20; Santa Cruz Biotechnology, Heidelberg, Germany), prepared in PBS with 2% Tween-20 and 2% normal donkey serum, overnight, at 4 °C. Secondary detection was performed with Alexa Fluor 568 donkey anti-goat conjugated secondary antibody (1:500, Life Technologies, Thermo-Fisher, Madrid, Spain). Finally, retinas were thoroughly washed in PBS and mounted with the RGC layer facing up and covered with anti-fading solution.

#### Retinal cryosections

The eyes were enucleated and post-fixed in 4% PFA for 1 h. Then, the cornea was carefully removed and the eyecup was fixed for an additional 1 h in 4% PFA. After washing in PBS, the tissue was cryopreserved in 15% sucrose in PBS for 1 h, followed by 30% sucrose in PBS for 1 h. The eyecups were embedded in tissue-freezing medium with 30% of sucrose in PBS (1:1), and stored at −80 °C. The tissue was sectioned on a cryostat (18 μm thickness) and the sections were mounted on Superfrost Plus glass slides.

Retinal slices were permeabilized with 0.1% Triton X-100 in PBS, followed by blockade with 10% normal goat serum and 1% bovine serum albumin (BSA) for 1 h. The slices were then incubated with primary antibodies, as follows: rabbit anti-Iba1 (1:1000; Wako, Osaka, Japan), rabbit anti-MHC-II (1:200; AbDSerotec, Oxford, UK), or mouse anti-GFAP (1:500; Merck Millipore, Billerica, MA, USA), in PBS with 1% BSA, overnight, at 4 °C. Secondary detection was performed with Alexa Fluor 568 goat anti-mouse and Alexa Fluor 488 goat anti-rabbit (all 1:500; Life Technologies, Carlsbad, USA). Nuclei were counterstained with DAPI (Life Technologies, Carlsbad, USA) and the slices were mounted with Glycergel (DAKO, Glostrup, Denmark).

The preparations were observed with a confocal microscope (LSM 710, Zeiss, Oberkochen, Germany) on an Axio Observer Z1 microscope using an EC Plan-Neofluar 40x/1.30 Oil DIC M27 objective.

### Transmission electron microscopy of optic nerves

Optic nerve samples were collected at approximately 1 mm from the optic chiasm and fixed with 2.5% glutaraldehyde in 0.1 M sodium cacodylate buffer (pH 7.2), supplemented with 1 mM calcium chloride for 2 h. Following rinsing in the same buffer, post-fixation was performed using 1% osmium tetroxide for 1 h. Samples were then washed in buffer and dehydrated in a graded ethanol series (30–100%), impregnated and embedded in Epoxy resin (Fluka Analytical, Sigma-Aldrich, St. Louis, MO, USA). For the evaluation of whole nerve and individual axons, ultrathin sections (70 nm) were mounted on copper grids (300 mesh) and stained with 2% uranyl acetate (15 min) and 0.2% lead citrate (10 min). Observations were carried out using a Tecnai G2 Spirit BioTWIN electron microscope (FEI) at 100 kV.

The electron microscopy images were graded in a masked-fashion by an independent observer for optic nerve axonal damage (Grade 1 = 0 to 25%, Grade 2 = 25 to 75%, Grade 3 = 75 to 100% of axons with myelin disarrangements). A second independent observer was asked to grade a subset of the images and κ-statistic was computed to measure inter-observer agreement. A strong inter-observer agreement was found (κ = 0.607, p < 0.001). The results were presented as the frequency distribution of each grade attributed to each animal.

### Image analysis

Retinal whole-mounts were examined and photographed with a microscope (Axioscop 2 Plus; Zeis, Oberkochen, Germany) equipped with a digital-high-resolution camera (ProgRes™ c10; Jenoptic, Jena, Germany) and a computer driven motorized stage (ProScan™ H128; Prior Scientific Instruments Ltd., Cambridge, UK) connected with an image analysis system (Image-Pro Plus 5.1 for Windows®; Media Cybernetics, Silver Spring, MD). Photomontages of the whole-mounts were constructed from 154 consecutive frames captured on the microscope side by side with no gap or overlap between them.

The individual images taken for each retinal photomontage were processed to assess the total number of FG^+^ cells and Brn3a^+^ cells in each retina with a specific cell counting subroutine to automatically count labeled cells in each frame, as previously described^40^,^62^.

Density of FG^+^ cells and Brn3a^+^ RGCs (cells/mm^2^) over the entire retinas were calculated and represented in isodensity maps, as described^40^.

### Real-time quantitative PCR

Total RNA was extracted from rat retinas using Trizol reagent. RNA samples were dissolved in 16 μL of Mili-Q water and total RNA concentration was determined using NanoDrop ND1000. Amplification of cDNA was performed according to the instructions provided by the manufacturer, using 1 μg of total RNA (NZYTech, Lisbon, Portugal). The resultant cDNA was treated with RNAse-H for 20 min, at 37 °C, and a 1:2 dilution was prepared for qPCR analysis. All samples were stored at −20 °C until analysis.

Genomic DNA contamination was assessed with a conventional PCR for β-actin using intron-spanning primers (Table 3), as described previously^63^. SYBR Green-based real-time quantitative PCR (qPCR) was performed using StepOnePlus, as previously described^15^. Ct values were converted to “Relative quantification” using the 2^−ΔΔCt^ method previously described^64^. Four candidate housekeeping genes (*Tbp*, *Hprt*, *Ywhaz* and *Rhodopsin*) were evaluated using NormFinder (a Microsoft Excel Add-in)^65^ and *Yhwaz* was identified as the most stable gene.

### TNF and IL-1β protein levels quantification by Enzyme-Linked Immunosorbent Assay (ELISA)

Protein levels of IL-1β and TNF were quantified by ELISA, according to the instructions provided by the manufacturer (Peprotech, London, UK). Briefly, total retinas were lysed in 20 mM imidazole-HCl, 100 mM KCl, 1 mM MgCl_2_, 1 mM EGTA, 1 mM EDTA, 10 mM NaF, 1% Triton X-100, supplemented with protease and phosphatase inhibitors (Roche, Basel, Switzerland). Then, lysates were sonicated and centrifuged at 16,000 g for 10 min at 4 °C and at 10,000 g for 5 min at 4 °C, respectively. The supernatant was collected and stored at −80 °C until use.

The cytokine concentration of each sample was normalized to the total protein concentration (determined by the bicinchoninic acid protein assay).

### Statistical analysis

The results are presented as mean ± standard error of the mean (s.e.m.). The normality of the data was assessed with Shapiro-Wilk normality test. The data were analyzed using Kruskall-Wallis test, followed by Dunn’s multiple comparison test, or Two-Way ANOVA followed by Tukey’s Multiple Comparison Test, as indicated in the figure legends. The statistical analysis was performed in Prism 6.0 Software for Mac OS X (GraphPad Software, Inc).

## Additional Information

**How to cite this article**: Madeira, M. H. *et al.* Caffeine administration prevents retinal neuroinflammation and loss of retinal ganglion cells in an animal model of glaucoma. *Sci. Rep.* **6**, 27532; doi: 10.1038/srep27532 (2016).

## Supplementary Material

Supplementary Information

## Figures and Tables

**Table 1:** Animal fluid intake and weight.

	Animals drinking water (n = 48)	Animals drinking caffeine (n = 49)
Fluid intake (mL/day)	23.7 ± 0.9	22.0 ± 0.7
Animal Weight (g)		
Day 0	200.1 ± 1.1	201.5 ± 1.7
Week 1	200.1 ± 1.1	201.5 ± 1.7
Week 2	234.4 ± 2.4	236.0 ± 1.8
Week 3	253.4 ± 1.9	250.8 ± 5.7

**Table 2:** Frequency distribution of optic nerves grading.

	Control		OHT		OHT + Caffeine	
	Animal 1	Animal 2	Animal 1	Animal 2	Animal 1	Animal 2
Grade 1	10	8	0	0	5	0
Grade 2	–	2	2	1	4	6
Grade 3	–	–	4	8	0	3

**Table 3:** Primers used in qPCR and RT-PCR.

Gene	GenBank number	Forward	Reverse	Amplicon Size (bp)
Adora 1	NM_017155.2	5′-TGAGTGTGGTAGAGCAAGAC-3′	3′-CAGACGAAGAAGTTGAAGTAGAC-3′	118
Adora2a	NM_053294	5′-GGCTATCTCTGACCAACA-3′	3′-TGGCTTGACATCTCTAATCT-5′	106
tnf	NM_012675	5′-CCCAATCTGTGTCCTTCT-3′	3′-TTCTGAGCATCGTAGTTGT-5′	90
il-1β	NM_031512	5′-ATAGAAGTCAAGACCAAAGTG-3′	3′-GACCATTGCTGTTTCCTAG-5′	109
mhc-ii	NM_013069.2	5′-CCACCTAAAGAGCCACTGGA-3′	3′-AGAGCTGGCTTCTGTCTTCAC-5′	101
Tspo	NM_012515.2	5′-TGTATTCGGCCATGGGGTATG-3′	3′-GAGCCAGCTGACCAGTGTAG-5′	105
trem2	NM_001106884.1	5′-AACTTCAGATCCTCACTGGACC-3′	3′-CCTGGCTGGACTTAAGCTGT-5′	90
yhwaz	NM_013011.3	5′-CAAGCATACCAAGAAGCATTTGA-3′	3′-GGGCCAGACCCAGTCTGA-5′	76
Actb	NM_031144	5′-GCTCCTCCTGAGCGCAAG-3′	3′-CATCTGCTGGAAGGTGGACA-5′	75
