# Supplementary material for: Caffeine administration prevents retinal neuroinflammation and loss of retinal ganglion cells in an animal model of glaucoma
Source: Sci Rep. 2016 Jun 8;6:27532. doi: 10.1038/srep27532 (PMC4897621; doi:10.1038/srep27532)
Supplement: Supplementary Information [file srep27532-s1.pdf]

SUPLLEMENTARY INFORMATION

<u>Manuscript:</u> Caffeine administration prevents retinal neuroinflammation and loss of retinal ganglion cells in an animal model of glaucoma

Maria H. Madeira, Arturo-Ortin-Martinez, Francisco Nadal-Nícolas, António F. Ambrósio, Manuel Vidal-Sanz, Marta Agudo-Barriuso, Ana Raquel Santiago

Supplementary figure 1

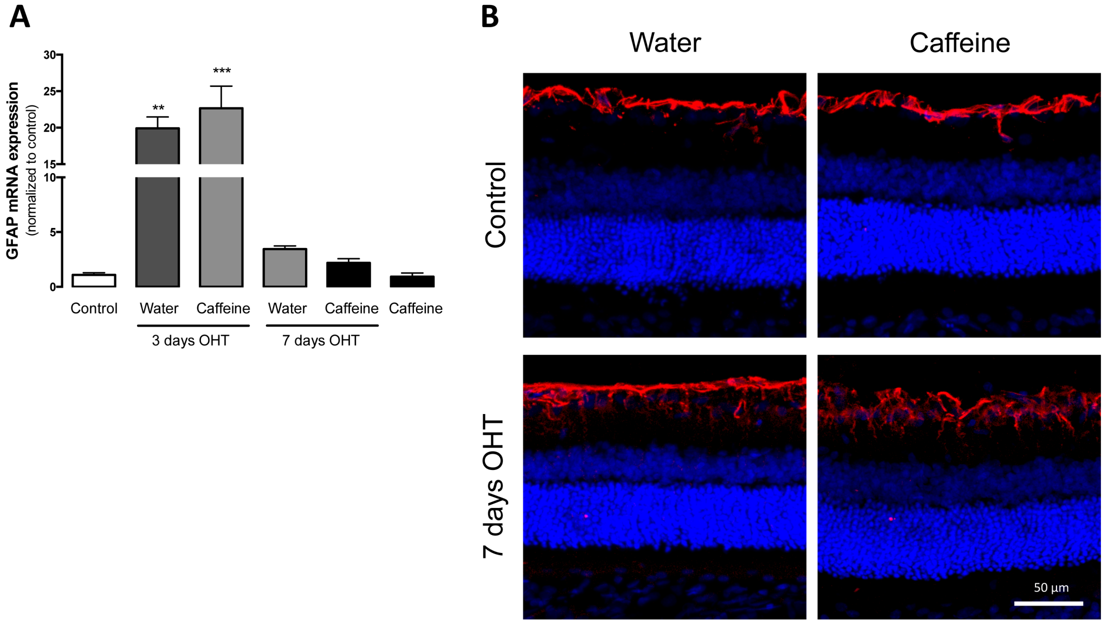

Supplementary figure 1 – Caffeine did not alter the effect of OHT in the expression of GFAP.

Retinal sections were immunostained for GFAP (marker of astroglial and Muller reactivity; red) and then imaged in a confocal microscope. Nuclei were stained with DAPI (blue). Representative images obtained from 3 independent experiments **(A).** Effects of caffeine administration on the mRNA expression of GFAP. Results are presented as fold change comparing with the control, from 5-7 independent experiments **(B)**. **P<0.01 and ***P<0.001, significantly different from control; Kruskall-Wallis test, followed by Dunn's multiple comparison test.
